# Enhancement of Phenolics, Resveratrol and Antioxidant Activity by Nitrogen Enrichment in Cell Suspension Culture of *Vitis* *vinifera*

**DOI:** 10.3390/molecules19067901

**Published:** 2014-06-11

**Authors:** Napaporn Sae-Lee, Orapin Kerdchoechuen, Natta Laohakunjit

**Affiliations:** School of Bioresources and Technology, King Mongkut’s University of Technology Thonburi, 49 Teintalay Rd., Thakam, Bangkhuntein, 10150 Bangkok, Thailand; E-Mails: na_shearer@hotmail.com (N.S.-L.); Nutta.lao@kmutt.ac.th (N.L.)

**Keywords:** phytochemical, ammonium nitrate, antioxidative activity, *trans*-resveratrol

## Abstract

Ammonium nitrate (NH_4_NO_3_), an important nitrogen source (34% N), has been used as an elicitor to stimulate plant growth and development as well as induce secondary metabolites under controlled conditions. In the present paper, we investigated the enhancement of cell biomass, total phenolics, resveratrol levels, and antioxidant activity of *Vitis vinifera* cv. Pok Dum by nitrogen enrichment (MS medium supplemented with NH_4_NO_3_ at 0, 500, 1,000, 5,000 and 10,000 mg/L). The highest accumulations of biomass, phenolics and resveratrol contents were observed at 8.8-fold (86.6 g DW/L), 15.9-fold (71.91 mg GAE/g DW) and 5.6-fold (277.89 µg/g DW) by the 14th day, in the medium supplemented with 500 mg/L NH_4_NO_3_. Moreover, the antioxidant activities of cultured grape cells estimated by the DPPH^●^ and ABTS^●+^ assay were positively correlated with phenolics and resveratrol, and the maximum activity was also observed in cultured cells with 500 mg/L NH_4_NO_3_ at 176.11 and 267.79 mmol TE/100 g DW, respectively.

## 1. Introduction

Secondary plant metabolites, which are biologically active non-nutrients and antioxidant constituents in plant material, have raised interest among scientists, food and drink manufacturers and consumers for their roles in the maintenance of human health [1]. Phenolics are broadly distributed in the plant kingdom and are the most abundant secondary metabolites of plants. Plant polyphenols have drawn increasing attention due to their potent antioxidant properties and their marked effects in the prevention of various oxidative stress-associated diseases such as cancer [2].

Grape (*Vitis vinifera*) fruit is a rich source of phenolics, in particular stilbenes, which have received intense focus due to their antioxidant properties with respect to cardioprotective effects and other health benefits [3]. In particular, great attention has been paid to the suggested health-promoting effects of *trans*-resveratrol (3,4',5-trihydroxy-*trans*-stilbene), a key natural product from red wine, that has attracted increasing attention around the world. In recent years, resveratrol and its derivatives (including its oligomers) have shown amazing chemical diversities and biological activities, and they have been emerging as promising new antioxidants [3].

The levels of phenolics and their derivatives in grapes vary due to several factors such as grape cultivars, agronomic conditions, and geographic regions. However, there is no standardized procedure to obtain phenolics- and resveratrol-enriched grapes that may be subsequently used to prepare necessary nutraceuticals for foods, cosmetics and pharmaceutical compounds [4]. The use of biotechnology is a particularly promising alternative to obtain valuable secondary metabolites under controlled conditions through plant cell cultures. *In vitro* cultures provide a source of highly active homogeneous cells that allow some plant limits such as slow growth, seasonal and environmental variations and diseases to be overcome [4]. Internally, macronutrients such as nitrogen play an important role not only in the growth of tissue cell lines, but also in the production of phenolic compounds in cell suspension cultures [5]. Nagella and Murthy [6] reported that 0.5 strength of MS medium NH_4_NO_3_ favored biomass accumulation while full strength NH_4_NO_3_ favored the maximum production of phytochemicals from cell suspension cultures of *Withania somnifera*. The concentration of 0.5 strength NH_4_NO_3_ of MS medium could enhance the highest biomass and production of gymnemic acid content from cell suspension cultures of *Gymnema sylvestre* [7]. The production of betacyanin in *Phytolacca americana* has increased in a high NO_3_^−^/NH_4_^+^ ratio medium [8]. Total nitrogen content is a contributing factor for controlling the pH of the growth media and it stimulates morphogenesis and embryogenesis, and thus is important for inducing callus formation in many plant cultures [5].

However, all the aforementioned effects of the culture medium differ from one species to another and from one compound to another. There are no reports on the effects of macronutrients of nitrogen (e.g., ammonium nitrate) on the biomass accumulation and resveratrol production in the cell suspension culture of *Vitis vinifera*. Therefore, establishing a suitable nitrogen concentration in the culture medium is a key step towards high production of secondary metabolites in plant cell and organ culture. The purpose of this study was to enhance phenolics, resveratrol and antioxidant activity by nitrogen enrichment of *Vitis vinifera* cv. Pok Dum cell suspension culture.

## 2. Results and Discussion

### 2.1. Effect of Ammonium Nitrate (NH_4_NO_3_) on Cell Growth

**Figure 1:**
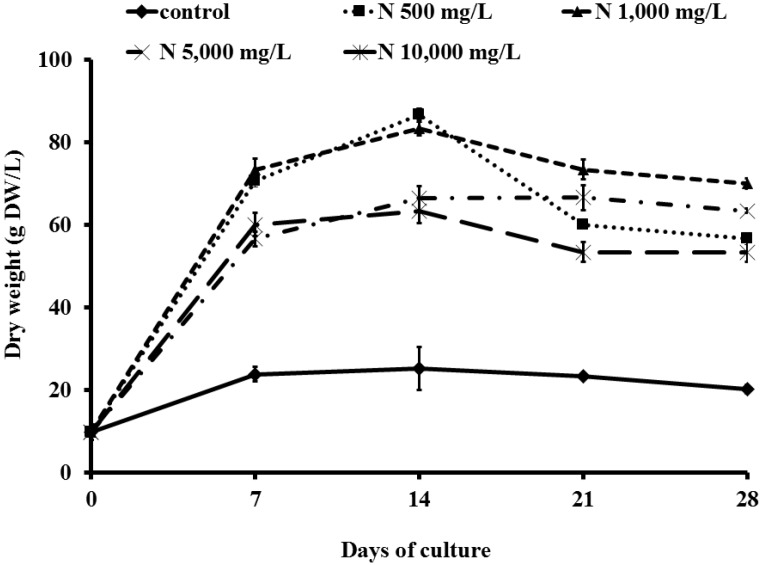
Growth of ‘Pok Dum’ *Vitis vinifera* cell suspension cultures after being treated with ammonium nitrate 0, 500, 1,000, 5,000 and 10,000 mg/L (control, N 500 mg/L, N 1,000 mg/L, N 5,000 mg/L and N 10,000 mg/L). Data are mean ± SD (*n* = 3).

Cell biomass of grape cell suspension cultures during the 28 days of culture ranged from 9.8 to 86.6 g DW/L (Figure 1). Cell growth of *V. vinifera* was in linear phase within 0–7 days, and then gradually increased until reaching a stationary phase within 7–21 days, followed by a death phase. The occurrence of the no-cells or death phase was due to consumption of the nutrients and a lack of oxygen [9]. Cell biomass was enhanced with an increase of NH_4_NO_3_ from 500 and 1,000 mg/L (*p* > 0.05). Cell dry weight of 500 mg/L NH_4_NO_3_ treated cells at day 7 and day 14 was increased 7.2- and 8.8-fold compared to the control. NH_4_NO_3_ 1,000 mg/L had similar stimulating effects on cell biomass, with 7.5- and 8.5-fold accumulations, compared with the control at day 7 and day 14 after the treatments, respectively. In contrast, grape cell treated with 5,000 and 10,000 mg/L NH_4_NO_3_ exhibited a lower rate of growth with only a 5.7- and 6.1-fold increase in dry weight after 7 days, and a 6.8- and 6.5-fold increase after 14 days, respectively. The highest dried biomass, 86.6 g DW/L, was found in 500 mg/L NH_4_NO_3_ treated cells on day 14 after treatment. This is due to the uptake of ammonium and nitrate ions by the cells, which are essential for their growth [9]. Results also showed that biomass of cells treated with low concentrations of NH_4_NO_3_ (500 and 1,000 mg/L) was greater than cells treated with high concentrations (5,000 and 10,000 mg/L). Like in research by Nagella and Murthy, our results showed that the growth of cell cultures might be inhibited by the addition of high concentration of NH_4_NO_3_ [6]. Ammonium is very diffusive and it easily accumulates into tissue, becoming very toxic if not immediately metabolized. When the ammonium concentration in the medium is low, most of the accumulated ammonium is metabolized by the cells. Whereas the ammonium concentration is excess, only a small part can be metabolized [10].

### 2.2. Effect of Ammonium Nitrate (NH_4_NO_3_) on Phenolic Content

**Figure 2:**
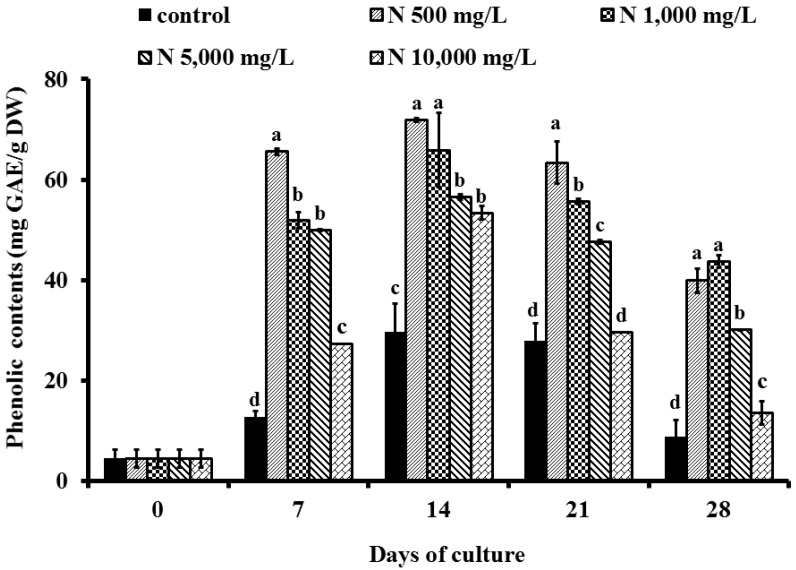
Effect of ammonium nitrate 0, 500, 1,000, 5,000 and 10,000 mg/L (control, N 500 mg/L, N 1,000 mg/L, N 5,000 mg/L and N 10,000 mg/L) on phenolic contents (mg GAE/g DW) of ‘Pok Dum’ *Vitis vinifera* cell suspension cultures. Data are mean ± SD (*n* = 3). Means at each day of culture with the same letter do not differ according to Duncan’s New Multiple Range Test, *p* ≤ 0.05.

In this study, stress response from five different concentrations of NH_4_NO_3 _on phenolic contents was monitored by the Folin-Ciocalteu method and expressed in gallic acid equivalents as shown in Figure 2. It was found that the phenolic contents began to increase rapidly during the early linear growth phase (7 days). The production of phenolic contents of treated cells with NH_4_NO_3_ at 500, 1,000, 5,000 and 10,000 mg/L increased more than those of the control. The highest amount of phenolics obtained in grape cells cultured in suspension containing 500 mg/L NH_4_NO_3_ on day 14 and 21. Results were in agreement with Zhong and Wang [11] who reported that lower concentrations of nitrogen resulted in the highest accumulation of ginsenoside in the *Panax quinquefolium* suspension cultures, while the high concentration (10,000 mg/L NH_4_NO_3_) decreased phenolics accumulation. 

The ammonium concentrations might have a direct or indirect effect on nitrate assimilation and secondary metabolite synthesis [10]. The 500 mg/L NH_4_NO_3_ enhanced the maximum value of phenolics in cultured grape cells on day 14 (71.91 mg GAE/g DW), or 15.9-fold as compared with the control cultured cells (day 0). However, the cultured grape cells produced fewer phenolics after 21 days of being treated with NH_4_NO_3_. Some results suggested that ammonium played an important role during the first few days of culture, *i.e.*, just before or during the rapid growth period [12]. This can be related to some observations by Bensaddek *et al.* [10] who showed that ammonium was totally and rapidly removed from the medium in the first days of culture preceding the exponential growth, while only a part of the nitrate was up taken, later and gradually. In 2001, Stewart *et al.* [13] demonstrated increased accumulation of phenylalanine ammonium lyase (PAL), the key enzyme of the phenylpropanoids pathway by abiotic stresses, including mineral nutrient stress. In particular, in nitrogen stress, there was an accumulation of phenolic compounds, such as flavonols, anthocyanins [13] and phenolic acids, including benzoic, cinnamic and coumarin derivatives [14]. Although nitrogen is an important inorganic nutrient for plants, being one of the major constituents of amino acids, proteins and nucleic acids, it is well known that nitrogen might affect plant growth and development, and induce a wide reprogramming of primary and secondary metabolism [15]. Moreover, the phenolic contents induced by nitrogen sources in cell suspension culture might be different from the levels found in grape seeds. Our previous research demonstrated that the phenolics in *V. vinifera* cv. Pok Dum seeds were 11% DW [16], whereas we detected significant effect of ammonium nitrate elicitation on generating phenolics, only 7% DW on day 14 of culturing. 

### 2.3. Effect of Ammonium Nitrate (NH_4_NO_3_) on Resveratrol Contents

**Figure 3:**
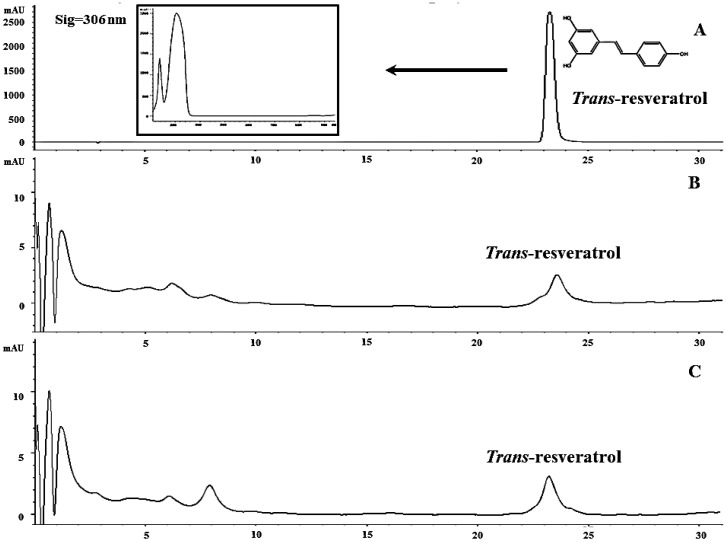
Chromatogram profile at 306 nm of (**A**) external standard *trans*-resveratrol with UV-Vis spectra, (**B**) 0 mg/L (control), and (**C**) 500 mg/L of *Vitis vinifera* cell treated with ammonium nitrate for 14 days.

**Figure 4:**
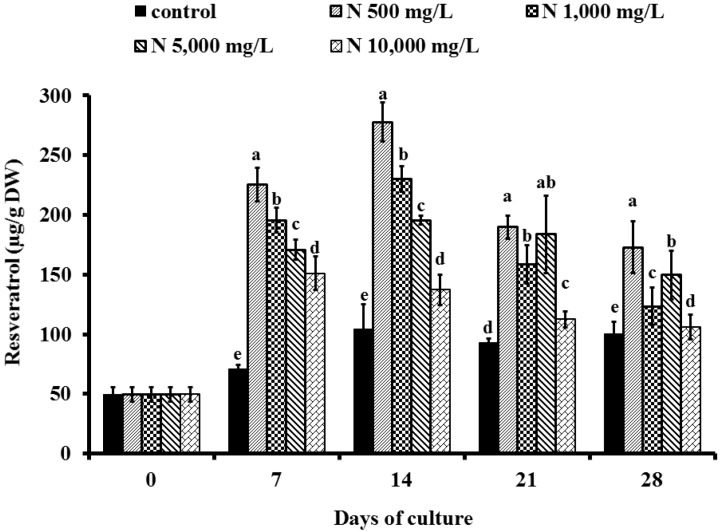
Effect of ammonium nitrate (control, N 500 mg/L, N 1,000 mg/L, N 5,000 mg/L and N 10,000 mg/L) on resveratrol contents (µg/g DW) of ‘Pok Dum’ *Vitis vinifera* cell suspension cultures. Data are mean ± SD (*n* = 3). Means at each day of culture with the same letter do not differ according to Duncan’s New Multiple Range Test, *p* ≤ 0.05.

To quantify the target molecules, *trans*-resveratrol was used as an external standard (Figure 3). The resveratrol content of *V. vinifera* cv. Pok Dum after treated with ammonium nitrate at 0, 7, 14, 21, and 28 days ranged from 49.63–277.89 µg/g DW (Figure 4). The resveratrol content in the treated cells rapidly increased and reached the maximum on 14 days. The accumulation of resveratrol contents by 500 mg/L NH_4_NO_3_ treated cells was greater than the control cells or treated cells with 1,000, 5,000 or 10,000 mg/L NH_4_NO_3_. The highest amount of resveratrol produced by grape cells cultured in suspension containing 500 mg/L NH_4_NO_3_ on day 14 was 277.89 µg/g DW. 

Although the resveratrol in grape cell suspension culture declined on day 21 and day 28, resveratrol of cells subjected to 500 mg/L NH_4_NO_3_ were greater than the other NH_4_NO_3_ treatments. A relatively similar graduation was previously observed for the white mulberry, which secondary metabolites have declined during steady stage of the growth responses. Nitrogen sources are important for secondary product synthesis of compounds such as alkaloids, anthocyanins, and shikonin from cell suspension cultures [17]. To our knowledge, cell cultures of *V. vinifera* have been investigated and focused on stimulating production of phenolic compounds and anthocyanin but there is no report on the stimulation of resveratrol metabolism in plant *in vitro* cultures by ammonium nitrate. This study represents the first report on ammonium nitrate, effecting on resveratrol production. Moreover, the production of resveratrol in this study was at the high level 0.028% DW or 277.89 mg/L, while analysis of *trans*-resveratrol production in untreated plant cell cultures was at a low level, less than 0.001% DW or 2–3 mg/L (data not shown).

### 2.4. Antioxidant Activities

#### 2.4.1. Determination of Free Radical Scavenging Activity Using 2, 2-Diphenyl-1-picrylhydrazyl (DPPH^●^) Assay

**Figure 5:**
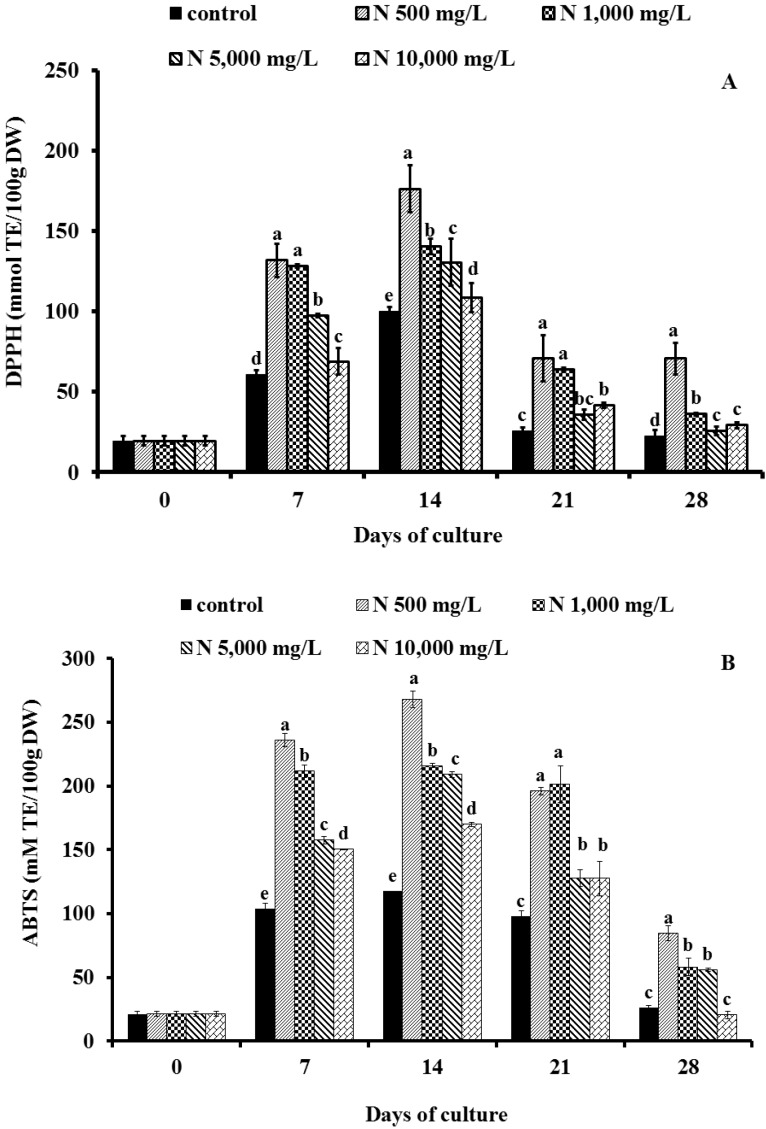
Effect of ammonium nitrate (control, N 500 mg/L, N 1,000 mg/L, N 5,000 mg/L and N 10,000 mg/L) on antioxidant activity measured by DPPH^•^ (**A**) and ABTS^•+^ (**B**) expressed in Trolox equivalents (mmol TE/100 g DW) of ‘Pok Dum’ *Vitis vinifera* cell suspension cultures. Data are mean ± SD (*n* = 3). Means at each day of culture with the same letter do not differ according to Duncan’s New Multiple Range Test, *p* ≤ 0.05.

The 2,2-diphenyl-1-picrylhydrazyl (DPPH^●^) activity is a proper indicator for investigating the free radical scavenging activities of phenolic compounds. When *V. vinifera* cells were cultured with five doses of NH_4_NO_3_, the antioxidant activity of treated cells increased in the early period from day 7 until day 14. The maximum activity was observed in the grape cells treated with NH_4_NO_3_ 500 mg/L on day 14, at which its scavenging activity by DPPH^●^ was 176.11 mmol TE/100 g DW (Figure 5A). However, the scavenging activity of cultured cells decreased when NH_4_NO_3_ in medium was provided up to 10,000 mg/L. In contrast to our observations, Cui *et al.* [18] reported that the antioxidant activities were reached to the maximum in the extracts of adventitious root of *Hypericum perforatum* suspension cultures with high nitrogen at 1,500–2,500 mg/L supplemented medium. A positive correlation was observed between DPPH radical scavenging activity and phenolic and resveratrol contents in cell suspension culture (*r^2^* = 0.6576 and 0.6756). 

Secondary metabolites, phenolic compounds in particular, are involved in plant responses to abiotic stresses and provide a significant contribution to the antioxidant activity of plant tissues. It is known that environmental stresses, including nitrogen excess or starvation, may increase the production of phenolic compounds [19]. In this study, nitrogen elicitors caused an increase of phenolics and resveratrol contents in the cultured *V. vinifera* cells, which the phenolics and resveratrol might be responsible for enhancing DPPH radical scavenging activity. 

#### 2.4.2. Determination of Free Radical Scavenging Activity Using the 2, 2'-Azinobis-(3-ethylbenzo-thiazoline-6-sulfonic acid) (ABTS^•+^) Assay

The effects of different concentrations of ammonium nitrate (0, 500, 1,000, 5,000 and 10,000 mg/L) on antioxidant activity as assessed by the ABTS radical cation (ABTS^•+^) in the *V. vinifera* cell suspension cultures are shown in Figure 5B. The antioxidant activity of grape cells cultured in suspension containing NH_4_NO_3_ increased from day 7 until day 14, and ranged from 21.21 to 267.79 mmol TE/100 g DW. The highest ABTS scavenging activity (267.79 mmol TE/100 g DW) was observed in the *V. vinifera* cells cultured in low levels of NH_4_NO_3_ medium (500 mg/L). This finding was in contrast with an observation in adventitious root extracts of *Hypericum perforatum* grown in the medium with high levels of nitrogen sources [18]. It was hypothesized that phenolics and resveratrol were efficient scavengers of free radicals [3]. The differences in antioxidant activity measured by different assays are likely to be due to the mechanism of action in these DPPH^•^ or ABTS^•+^ assays. In this study, ABTS^•+^ method is a more sensitive to determine antioxidative capacity of phenolic compounds samples, because it can determine the capacity at low inhibitor concentrations. The greater radical-scavenging activities detected by ABTS^•+ ^than DPPH^•^ were similar to the result of *Habenaria edgeworthii* callus suspension cultures [20], but the radical-scavenging activity of the grape cells cultured in suspension containing NH_4_NO_3_ was greater than that from *V. vinifera* cell cultures as reported by Teguo *et al*. [21].

## 3. Experimental

### 3.1. Plant Materials

In this study, red grape cv. Pok Dum was chosen due to its high phenolics, resveratrol level, and antioxidant activity, as reported in our earlier study [16]. Young leaf explants from grape seedlings cv. Pok Dum were soaked in 70% alcohol for 30 s, then in 5% NaOCl for 3 min and 2% NaOCl for 5 min, followed by three sequential rinses for 5 min in sterile distilled water.

### 3.2. Callus Induction and Subculture

To induce callus formation, sterile leaf explants excised from *V. vinifera* cv Pok Dum seedlings were cultured in MS medium, which consisted of 4% agarized MS medium supplemented with 20 mg/L sucrose as a source of carbon and 0.1 mg/L 6-benzyladenine (BA) as cytokinin. The initial pH of the medium was adjusted to 5.8 before autoclaving. The cultures were incubated at 25 ± 1 °C with a 16 h light (108 µmol m^−2^ s^−1^) and an 8 h dark photoperiod by means of 40 W cool-white fluorescent tubes (Phillips, Kolkata, India). The callus was subcultured every 14 days.

### 3.3. Cell Suspension Culture

Cell suspension cultures were initiated from the 4-week-old callus of *V. vinifera* cv Pok Dum by transferring 2 g friable callus into a 250 mL Erlenmeyer flask containing 50 mL of MS liquid medium having the same formulations as the callus cultures except agarized. Cell suspension cultures were maintained on a rotary shaker at 110 rpm at 25 ± 1 °C for 28 days. To stimulate phenolics and resveratrol production from cell suspensions, five different concentrations of 0, 500, 1,000, 5,000, and 10,000 mg/L of ammonium nitrate (NH_4_NO_3_) (Sigma-Aldrich, ≥98%) of a nitrogen source were treated, incorporating with full-strength MS medium at 1,650 mg/L of ammonium nitrate (as control or 0 mg/L of treated NH_4_NO_3_). The experimental design was a randomized complete block design (RCBD) with three replications. Sample cells were collected immediately (time 0) and at 7, 14, 21, and 28 days after treatment.

### 3.4. Determination of Cell Biomass

Every 7 days, nine flasks of each cell line were harvested by filtration through Whatman filter paper (No. 1) under a vacuum. Dry weights (DW) of treated cells were recorded after drying at 60 °C in an oven until reach a constant weight.

### 3.5. Determination of the Total Phenolic and Resveratrol Contents

Dried cell aggregates separated from the culture medium by vacuum filtration were ground and steeped in 1% (v/v) HCl methanol solution for 16 h. The volume of 1% (v/v) HCl methanol solution was 50 times of the sample weight. Samples were centrifuged at 10,000 rpm for 10 min. Supernatants were used for identification of phenolic compounds and resveratrol. 

#### 3.5.1. Determination of Total Phenolics

The amount of total phenolics in all extracts was determined by the Folin-Ciocalteu method [20] using gallic acid as the standard. Distilled water (0.95 mL) was combined with 50 µL of sample, 5 mL of 10% Folin-Ciocalteu’s reagent and 4 mL of 7.5% sodium carbonate (Na_2_CO_3_). The mixture was vortexed thoroughly and, after incubation at 40 °C for 30 min, the absorbance was measured at 765 nm against a blank without the sample using a UV-Visible Spectrophotometer (Thermo Fisher Scientific, Madison, WI, USA). Quantification was done on the basis of the standard curve of gallic acid (solution of gallic acid in 20% ethanol, 10–50 mg/L). Results were expressed as mg of gallic acid equivalents (GAE) per gram dry weight (mg GAE/g DW) of the sample.

#### 3.5.2. Determination of *Trans*-Resveratrol

The quantification of *trans*-resveratrol in grape cell suspension culture samples was carried out on an Agilent 1200 HPLC system (Waldbronn, Germany) equipped with an Agilent 1200 series DAD detector and autosampler. Separation was achieved using a ZORBAX Eclipse XDB-C18 column (150 mm × 4.6 mm id, 5 µm packing; Agilent, Santa Clara, CA, USA), with a precolumn of the same material; the column temperature was maintained at 40 °C. The HPLC conditions were described previously [17]. In summary, the elution profile was as follows: 0 min, 83.5% A, 16.5% B; 13 min, 82.0% A, 18.0% B; 15 min, 82.0% A, 18.0% B; 17 min, 77.0% A, 23.0% B; 21 min, 75.0% A, 25.0% B; 27 min, 68.5% A, 31.5% B; 30 min, 0% A, 100% B, where solvent A was glacial acetic acid in water (52.6:900 v/v) and solvent B was 20% phase A and 80% acetonitrile at a flow rate of 1.0 mL/min. Identification of *trans*-resveratrol was carried out by comparison of the retention time of standard and that within the extracts. Calibration curves were plotted from 0.005 to 10 mg/mL. The samples (100 µL) were directly injected after filtration through a 0.45 µm membrane filter. A photodiode array detector was used and quantification was done at 306 nm for *trans*-resveratrol.

### 3.6. Measurement of Antioxidant Activities

#### 3.6.1. 1,1-Diphenyl-2-picrylhydrazyl (DPPH^●^) Assay

The traditional DPPH^•^ assay [20] was modified for this study. DPPH (0.1 mmol) was prepared in 80% ethanol (v/v) and 3 mL was mixed with 1 mL of sample extract and allowed to stand in the dark (22 ± 1 °C, 30 min). The reduction in the absorbance at 517 nm was recorded using a UV-Visible spectrophotometer (Thermo Fisher Scientific) and results were expressed in mmol Trolox equivalents (TE) per 100 g dry weight (mmol TE/100 g DW) of the sample. 

#### 3.6.2. 2,2-Azinobis-(3-ethylbenzothiazoline-6-sulfonic acid) (ABTS^•+^) Assay

Total antioxidant activity was measured by the 2, 2-azinobis (3-ethylbenzothiazoline-6-sulfonic acid) radical scavenging (ABTS^•+^) method [20] with minor modifications. In brief, 7.0 mmol ABTS and 2.45 mmol potassium persulfate were mixed for the production of ABTS cation (ABTS^•+^) and kept in the dark (12 h, 22 ± 1 °C). ABTS solution was diluted with 80% (v/v) ethanol until an absorbance of 0.70 (±0.05) was obtained at 734 nm. For sample analysis, 4 mL of diluted ABTS^•+^ solution was added to 1 mL of methanolic extract and mixed thoroughly. The reaction mixture was allowed to stand (22 ± 1 °C, 6 min, dark) and then the absorbance was recorded in a UV-Visible Spectrophotometer (Thermo Fisher Scientific) at 734 nm. For each antioxidant assay, a Trolox aliquot was used to develop a 50–500 mmol standard curve. All data were expressed in mmol Trolox equivalents (TE) per 100 g dry weight (mmol TE/100 g DW) of the sample. 

### 3.7. Statistical Analysis

Data were collected with respective intervals (day 0, 7, 14, 21, and 28) and presented as mean ± SD (*n* = 3) of each independent treatment. The statistical analysis was performed using SAS software for PC Version 6 (SAS Institute, Cary, NC, USA). The data were analyzed using a general linear model for analysis of variance. Significant differences between individual treatments were determined using a Duncan’s New Multiple Range Test (DMRT).

## 4. Conclusions

We have demonstrated that treatment with ammonium nitrate in *V. vinfera* cv. Pok Dum cell suspensions could induce phenolics and resveratrol production. The culture conditions in this study were suitable for biomass, phenolics and resveratrol production of *V. vinifera* cv. Pok Dum cell suspensions. The highest accumulation of biomass was recorded in grape cells cultured in the medium with 500 mg/L NH_4_NO_3_. Phenolic and resveratrol contents of cultured grape cells were the highest at 15.9- and 5.6-fold (compared to those of day 0) in suspension containing 500 mg/L NH_4_NO_3_. The maximum antioxidant activity of treated grape cells was also recorded in the same medium. These results represent a further step towards the use of grape cell cultures in fed-batch bioreactors as a promising alternative to whole plant extraction for the industrial production of plant phenolics. 

## References

[1] Cherif A.O., Rasooli I. (2012). Phytochemicals components as bioactive foods. Bioactive Compounds in Phytomedicine.

[2] Dai J., Mumper R.J. (2010). Plant phenolics: Extraction, analysis and their antioxidant and anticancer properties. Molecules.

[3] He S., Yan X. (2013). From resveratrol to its derivatives: New sources of natural antioxidant. Curr. Med. Chem..

[4] Ferri M., Dipalo S.C.F., Bagni N., Tassoni A. (2011). Chitosan elicits mono-glucosylated stilbene production and release in fed-batch bioreactor cultures of grape cells. Food Chem..

[5] Narayan M.S., Venkataraman L.V. (2002). Effect of sugar and nitrogen on the production of anthocyanin in cultured carrot (*Daucus carota*) cells. J. Food Sci..

[6] Nagella P., Murthy H.N. (2010). Establishment of cell suspension cultures of *Withania somnifera* for the production of withanolide A. Bioresour. Technol..

[7] Praveen N., Murthy H.N., Chung I.M. (2011). Improvement of growth and gymnemic acid production by altering the macro elements concentration and nitrogen source supply in cell suspension cultures of *Gymnema sylvestre* R. Br. Ind. Crop Prod..

[8] Lee Y., Lee D.-E., Lee H.-S., Kim S.-K., Lee W.-S., Kim S.-H., Kim M.-W. (2010). Influence of auxins, cytokinins, and nitrogen on production of rutin from callus and adventitious roots of the white mulberry tree (*Morus alba* L.). Plant Cell Tiss. Org..

[9] Gueven A., Knorr D. (2011). Isoflavonoid production by soy plant callus suspension culture. J. Food Eng..

[10] Bensaddek L., Gillet F., Saucedo J.E.N., Fliniaux M.-A. (2001). The effect of nitrate and ammonium concentrations on growth and alkaloid accumulation of *Atropa belladonna* hairy roots. J. Biotechnol..

[11] Zhong J.J., Wang S.J. (1998). Effects of nitrogen source on the production of ginseng saponin and polysaccharide by cell cultures of *Panax quinquefolium*. Process Biochem..

[12] Amdoun R., Khelifi L., Khelifi-Slaoui M., Amroune S., Benyoussef E.-H., Thi D.V., Assaf-Ducrocq C., Gontier E. (2009). Influence of minerals and elicitation on *Datura stramonium* L. tropane alkaloid production: Modelization of the *in vitro* biochemical response. Plant Sci..

[13] Stewart A.J., Chapman W., Jenkins G.I., Graham I., Martin T., Crozier A. (2001). The effect of nitrogen and phosphorous deficiency on flavonol accumulation in plant tissues. Plant Cell Environ..

[14] Kovacik J., Klejdus B., Backor M., Repcak M. (2007). Phenylalanine ammonialyase activity and phenolic compounds accumulation in nitrogen-deficient *Matricaria chamomilla* leaf rosettes. Plant Sci..

[15] Scheible W.-R., Morcuende R., Czechowski T., Fritz C., Osuna D., Palacios-Rojas N., Schindelasch D., Thimm O., Udvardi M.K., Stitt M. (2004). Genome-wide reprogramming of primary and secondary metabolism, protein synthesis, cellular growth processes, and the regulatory infrastructure of Arabidopsis in response to nitrogen. Plant Physiol..

[16] Khunthajaroen T., Kerdchoechuen O., Laohakunjit N. (2011). Resveratrol, catechin and antioxidant activity from ‘Pok Dum’ grape extracts. J. Agric. Sci..

[17] Sakuta M., Takagi T., Komamine A. (1987). Effects of nitrogen source on betacyanin accumulation and growth in suspension cultures of *Phytolacca americana*. Physiol. Plantarum..

[18] Cui X.-H., Murthy H.N., Wu C.-H., Paek K.-Y. (2010). Adventitious root suspension cultures of *Hypericum perforatum*: Effect of nitrogen source on production of biomass and secondary metabolites. In Vitro Cell. Dev.-Pl..

[19] Kovacik J., Backor M. (2007). Changes of phenolic metabolism and oxidative status in nitrogen-deficient *Matricaria chamomilla* plants. Plant Soil.

[20] Giri L., Dhyania P., Rawata S., Bhatta I.D., Nandia S.K., Rawala R.S., Pandeb V. (2012). *In vitro* production of phenolic compounds and antioxidant activity in callus suspension cultures of* Habenaria edgeworthii*: A rare Himalayan medicinal orchid. Ind. Crop Prod..

[21] Teguo P.W., Fauconneau B., Deffieux G., Huguet F., Vercauteren J., Merillon J.M. (1998). Isolation, identification, and antioxidant activity of three stilbene glucosides newly extracted from *Vitis vinifera* cell cultures. J. Nat. Prod..

